# The Development of *Plasmodium falciparum*-Specific IL10 CD4 T Cells and Protection from Malaria in Children in an Area of High Malaria Transmission

**DOI:** 10.3389/fimmu.2017.01329

**Published:** 2017-10-19

**Authors:** Michelle J. Boyle, Prasanna Jagannathan, Katherine Bowen, Tara I. McIntyre, Hilary M. Vance, Lila A. Farrington, Alanna Schwartz, Felistas Nankya, Kate Naluwu, Samuel Wamala, Esther Sikyomu, John Rek, Bryan Greenhouse, Emmanuel Arinaitwe, Grant Dorsey, Moses R. Kamya, Margaret E. Feeney

**Affiliations:** ^1^Department of Medicine, University of California San Francisco, San Francisco, CA, United States; ^2^Center for Biomedical Research, The Burnet Institute, Melbourne, VIC, Australia; ^3^Department of Medicine, Stanford University, Stanford, CA, United States; ^4^Infectious Diseases Research Collaboration, Kampala, Uganda; ^5^Department of Medicine, Makerere University College of Health Sciences, Kampala, Uganda; ^6^Department of Pediatrics, University of California San Francisco, San Francisco, CA, United States

**Keywords:** *Plasmodium falciparum*, CD4 T cells, malaria, tolerance, interleukin 10

## Abstract

Cytokine-producing CD4 T cells have important roles in immunity against *Plasmodium falciparum (Pf)* malaria. However, the factors influencing functional differentiation of *Pf-*specific CD4 T cells in naturally exposed children are not well understood. Moreover, it is not known which CD4 T-cell cytokine-producing subsets are most critical for protection. We measured *Pf-*specific IFNγ-, IL10-, and TNFα-producing CD4 T-cell responses by multi-parametric flow cytometry in 265 children aged 6 months to 10 years enrolled in a longitudinal observational cohort in a high malaria transmission site in Uganda. We found that both age and parasite burden were independently associated with cytokine production by CD4 T cells. IL10 production by IFNγ^+^ CD4 T cells was higher in younger children and in those with high-parasite burden during recent infection. To investigate the role of CD4 T cells in immunity to malaria, we measured associations of *Pf*-specific CD4 cytokine-producing cells with the prospective risk of *Pf* infection and clinical malaria, adjusting for household exposure to *Pf*-infected mosquitos. Overall, the prospective risk of infection was not associated with the total frequency of *Pf-*specific CD4 T cells, nor of any cytokine-producing CD4 subset. However, the frequency of CD4 cells producing IL10 but not inflammatory cytokines (IFNγ and TNFα) was associated with a decreased risk of clinical malaria once infected. These data suggest that functional polarization of the CD4 T-cell response may modulate the clinical manifestations of malaria and play a role in naturally acquired immunity.

## Introduction

Despite a decline in the incidence of malaria globally, over 50% of the world’s population remains at risk of infection (1), with the largest burden caused by *Plasmodium falciparum (Pf)* parasites. Our incomplete understanding of the mechanisms mediating naturally acquired immunity to *Pf* poses a barrier to the development of vaccines and other control strategies. In endemic settings, the incidence of symptomatic malaria declines with increasing age and cumulative exposure. However, even highly exposed individuals remain susceptible to asymptomatic *Pf* infection into adulthood, and adults do not differ in time-to-infection compared with children, indicating that sterile immunity does not usually develop in response to natural infection (2). It is likely that the biological mechanisms underlying protection from infection and restriction of parasite growth are distinct from those that mediate protection from symptomatic malaria.

CD4 T-cell responses are important components of naturally acquired and vaccine-induced immunity to malaria [reviewed in Ref. (3)]. However, many aspects of the *Pf-*specific CD4 T-cell response in naturally exposed individuals, including the critical effector functions responsible for preventing infection and clinical malaria, remain poorly understood. There has been a considerable body of research assessing the role of cytokines in protection or risk of malaria, which has highlighted a role for several cytokines including interferon gamma (IFNγ), interleukin 10 (IL10), and tumor necrosis factor alpha (TNFα) in anti-malarial immunity [reviewed in Ref. (4–6)]. However, these studies have largely been based on plasma cytokine measurements or, alternatively, measurement of cytokine production by bulk cell populations (e.g., ELISA of culture supernatants), and have therefore been unable to characterize the cytokines (or combinations of cytokines) that are produced at the individual cell level, or to phenotype the cell producing the cytokines (i.e., CD4 vs. CD8 T cells, vs. others, such as γδ T cells). This is of fundamental importance because antigen-specific T cells are functionally heterogeneous, and the functional polarization of CD4 T cells, as defined by their cytokine production profiles, has been associated with the ability––or failure––to prevent or contain infection in several infectious disease models. In the few published studies which have used multicolor flow cytometry to assess *Pf-*specific cytokine production by CD4 T cells at an individual cell level, it has been shown that the *Pf-*specific CD4 T-cell response in highly malaria-exposed children is dominated by IFNγ and IL10 coproducing cells (7–10). Interleukin 10 production by CD4 T cells may help to control immunopathology and limit symptoms of infection (10), as suggested by murine models of malaria and other parasitic infections such as *Leishmania* and toxoplasmosis, where IL10 production from T helper cell (Th1) IFNγ-producing CD4 T cells is essential for dampening inflammatory responses, preventing disease (5, 11, 12), and protecting from IFNγ-dependent, TNFα-mediated pathology (13). CD4 T-cell responses may also be important in mediating protection from malaria by preventing parasite infection or restricting parasite replication once infection is established. We have recently shown that in areas of high malaria transmission, inflammatory CD4 T cells producing IFNγ and TNFα dominate in immune adults, who rarely experience high-density infections or clinical malaria despite constant exposure to infection (9).

Here, we studied a large and well-characterized longitudinal cohort of children from a highly malaria-endemic region of Eastern Uganda (14). We quantified *Pf-*specific IFNγ-, IL10-, and TNFα-producing CD4 T cells from 265 children aged 6 months to 10 years, and identified factors that influence the functional differentiation of *Pf-*specific CD4 T cells. Then, using detailed clinical outcome measures and household-level mosquito data to control for heterogeneity in environmental exposure to infected mosquitos (15), we analyzed the relationship between these CD4 subsets and prospective protection from *Pf* infection, and (separately) risk of developing clinical malaria once infected. Our data suggest opposing roles for TNFα and IL10 *Pf-*specific CD4 T cells in naturally acquired immunity to malaria in children.

## Materials and Methods

### Ethics Statement

This study was carried out in accordance with the recommendations of Uganda National Council of Science and Technology and the institutional review boards of the University of California, San Francisco, and Makerere University, with written informed consent from all adult subjects or parent/guardian of all study participants. All subjects gave written informed consent in accordance with the Declaration of Helsinki.

The protocol was approved by the institutional review boards of the University of California, San Francisco (institutional review board number 11-05995; reference number 067647), and Makerere University.

### Study Participants

Samples were obtained from participants in the East African International Centers of Excellence in Malaria Research “PRISM” Nagongera study cohort that was initiated in 2011 and is ongoing (14). This cohort consists of 100 households within the Nagongera sub-county in Tororo district. Malaria transmission in this region is holoendemic, with slight increases in malaria incidence and parasite prevalence during two annual seasonal peaks from October to January and April to July. In all households, one adult caregiver and all eligible children aged 6 months to 10 years were enrolled in the study. Exclusion criteria included any known chronic medical conditions requiring specialized care (including HIV infection). The cohort is dynamic, with children exiting the study at age 11, and new children born into study households enrolled at 6 months of age. For the work presented here, all children were scheduled for an “immunology blood draw” with samples taken between January and April 2013. Children who presented with malaria (currently febrile and blood smear detectible parasites), or had a recorded episode of malaria in the last 7 days, were re-scheduled to be bled at a later time point. In total, plasma blood mononuclear cell (PBMC) responses from 265 out of 270 children enrolled at the time of sampling were collected and analyzed. The study cohort characteristics of sampled children are presented in Table 1.

**Table 1:** Study cohort characteristics.

Characteristic	Finding
Number of children	265
Age, years (median, IQR)	5.8 (3.5–8.1)
Patent infection at blood draw (blood smear positive)	77 (29%)
Subpatent infection at blood draw (PCR positive, smear negative)	29 (11%)
Any infection at blood draw (blood smear and/or PCR positive)^a^	106 (40%)
Clinical characteristics in the year prior to blood draw	
Person-days observed (median, min–max)	365 (36–365)
Malaria incidence (median, IQR)	2.0 (1.0–4.0)
Parasite density during infection in last 3 months (log_10_, parasite/μL, median, IQR)^b^	3.9 (3.3–4.6)
Characteristics in the year following blood draw	
Number of routine visits/child^c^ (median, min–max)	4 (0–5)
Odds of any infection at routine visit^d^ (OR, 95% CI)	4.8 (3.6–6.5)
Odds of malaria at routine visit (OR, 95% CI)	0.26 (0.21–0.33)
dMER in year following blood draw, female Anopheles/house/day (median IQR)^e^	27.3 (16.5–37.1)

*^a^Current infection measured by PCR from dried blood spots and/or blood smear, all infections are asymptomatic*.

*^b^Parasite density is the most recent patent, blood smear positive parasite infection within the last 3 months, log transformed. In total, 211 children had a patent infection in 3 months prior to blood draw*.

*^c^For protection analysis, all routine visits were considered. Routine visit was characterized as no-infection, low-density infection (LAMP positive, blood smear negative), patent infection (blood smear positive), or malaria (blood smear positive and fever at visit, or within 21 days prior and 7 days following visit). Odds of infection were calculated with mixed-effects logistic regression*.

*^d^Odds of any infection at routine includes symptomatic malaria infection cases, and asymptomatic patent (blood smear detectable) or asymptomatic subpatent (blood smear negative, LAMP positive)*.

*^e^dMER is estimated from the mean household CDC light trap counts of Anopheles mosquito measured at monthly intervals over the 12 months following blood draw*.

*dMER, daily mosquito exposure rate; LAMP, loop-mediated isothermal amplification; PCR, polymerase chain reaction*.

### Clinical Management and Measurement of Clinical Parameters

Upon enrollment all study participants were given an insecticide treated bed net and followed for all medical care at a dedicated study clinic. All participants were reimbursed for travel to and from clinic for all visits, increasing the likelihood that all cases of malaria infection were detected. Furthermore, participants agreed to avoid all antimalarial medications administered outside the study, and compliance was tracked with questionnaire at all routine study visits. Children who presented with a fever (tympanic temperature ≥38.0°C) or history of fever in the previous 24 h had blood obtained by finger prick for a thick smear. If the thick smear was positive for *Plasmodium* parasites, the patient was diagnosed with malaria regardless of parasite density, and treated with artemether–lumefantrine, the recommended treatment for malaria in Uganda. Incident episodes of malaria were defined as all febrile episodes accompanied by any parasitemia requiring treatment, but not preceded by another treatment in the prior 14 days.

Routine assessments with active case detection were performed in the study clinic every 3 months, including blood smears and dry blood spots to detect for parasite infection. Negative blood smears obtained at routine assessments were tested for the presence of submicroscopic malaria parasites using loop-mediated isothermal amplification (LAMP) (16). At the time of routine assessments, children were divided into four categories: (1) no evidence of parasite infection; (2) asymptomatic, submicroscopic (LAMP positive) infection; (3) asymptomatic, blood smear positive infection, or (4) symptomatic malaria, with a window of 21 days prior to and 7 days following the routine visit to ensure capture of malaria episodes that were recently treated or infections that soon became symptomatic.

To assess for submicroscopic infection at the time of the “immunology” blood draw, *Plasmodium* DNA was extracted from dried blood spots and analyzed by polymerase chain reaction (PCR) as previously described (17). In 17 children (6.3%), PCR was not performed due to missing dried blood spots and current infection status was imputed from blood smear (10 were positive by blood smear, and 7 were negative). All analysis considered infection status as positive if participant had either a blood smear patent infection, or a PCR positive, blood smear negative subpatent infection.

### Daily Mosquito Exposure Rate (dMER)

The dMER was calculated for each individual based on the mean household-level female Anopheles mosquito counts obtained from CDC light traps placed overnight (once per month) within the household of each individual trial participant (15). For analyses to assess drivers of CD4 T-cell responses, dMER was calculated as the mean female Anopheles mosquito counts from the prior 12 months, and for analyses to assess outcomes of protection, dMER was calculated as the mean female Anopheles mosquito counts from the 12 months following blood draw. dMER was used in analysis as a categorical variable (0–8, >8–40, >40–80, and >80 mosquitos/household/day), based on analyses of the relationship between dMER and infection (Table S1 in Supplementary Material).

### Measuring *Pf* CD4 T-Cell Responses

Analysis of CD4 T-cell responses to *Pf*-infected red blood cells (RBCs) *via* intracellular cytokine staining was performed as previously described (8, 9). PBMCs were stimulated with *Pf-*infected RBCs or uninfected RBCs and CD4 T-cell production of IFNγ, IL10, and TNFα were measured *via* intracellular staining. PBMCs were thawed using standard methods and rested overnight in 10% fetal bovine serum. A total of 10^6^ PBMCs were stimulated with intact purified trophozoite/schizont-stage *Pf* (clone 3D7)-infected RBCs or uninfected RBCs at an effector to target ratio of 1:2. Parasite cultures were routinely confirmed mycoplasma negative. Following 6 h of stimulation, Brefeldin A and monensin (BD Pharmingen) were added (10 µg/mL). At 24 h, cells were washed, and surface and intracellular staining was performed with the following antibodies: from BD Pharmingen, anti-CD3-PerCP (SK7), anti-CD8-APC-H7 (SK1), anti-IFNγ-PE-Cy7 (B27), anti-IL-10-PE (JES3-19F1), and anti-TNFα-FITC (6401.1111); from Biolegend, anti-CD4-BV650 (OKT4), anti-CD14-Alexa700 (M5E2), anti-CD19-Alexa700 (HIB19), and anti-T-Bet BV421 (4B10); from Milteny anti-γδVδ2-APC (123R3); from R&D anti-BLIMP-1 Alexa647 (646702); and from Invitrogen, Live/Dead aqua amine. Samples were acquired on a BD LSR2 flow cytometer with FACSDiva. A median of 121,000 CD4 T cells were collected (IQR 98,310–144,000, minimum 10,000, maximum 197,000).

Flow cytometry data were analyzed using FlowJo software (Tree Star, San Carlos, CA, USA) and Pestle (version 1.7)/Spice (version 5.3; M. Roederer, Vaccine Research Center, National Institute of Allergy and Infectious Diseases, National Institute of Health available at: http://exon.niaid.nih.gov (18). Color compensation was performed using single-color cell controls or beads stained for each fluorochrome. Responding cells were gated as lymphocytes/singlets/CD14^−^CD19^−^Aqua^−^/CD3^+^γδVδ2^−^/CD8^−^/CD4^+^. Cytokine production was gated as CD4 producing IFNγ, IL-10, or TNFα, and Boolean gating was performed to categorize cells into subsets (Figure S1 in Supplementary Material). To calculate frequencies of *Pf* -specific CD4 T cells, background responses to uninfected RBCs were subtracted from each subset (Figure S2 in Supplementary Material). For data analysis, IFNγ^+^/IL10^+^/TNFα^+^ and IFNγ^+^/IL10^+^/TNFα^+^ responses were combined. Data were analyzed as the frequency of each subset as % of total CD4 T cells. To assess the composition of the *Pf-*specific response, total IFNγ, IL10, or TNFα frequencies or specific subsets as enumerated *via* Boolean gating were divided by the frequency of total responding CD4^+^ T cells and multiplied by 100 to calculate the % of total response.

### Statistical Data Analysis

All statistical methods were performed using Prism 4 (GraphPad), STATA version 12 (College Station), or SPICE v.5.3 version 5.3; M. Roederer, Vaccine Research Center, National Institute of Allergy and Infectious Diseases, National Institute of Health available at: http://exon.niaid.nih.gov) (18). Associations between dMER and age with probability of infection were calculated using Spearman’s correlations and linear regression analysis. To assess relationships between age and dMER with retrospective odds of infection measured at quarterly routine assessments, the odds of any infection (defined as either LAMP positive or blood smear positive with or without symptoms) were calculated using multilevel mixed-effects logistic regression, accounting for repeat measures within individuals and clustered on household to account for multiple children in each individual household.

For CD4 T-cell responses, frequencies and proportions of responses were compared with Wilcoxon rank sum for binary variables, Kruskal–Wallis for group variables (age groups: 0–3, >3–7, >7 years), and *via* Spearman’s rank correlation for continuous variables. For multivariate analysis, associations were made *via* linear regression, with the frequencies of CD4 T-cell responses log transformed, and predictor variables age (linear), current PCR positive infection (dichotomous), and dMER (categorical 0–8, >8–40, >40–80, >80 female *Anopheles*/house/day) assessed cocurrently where indicated. To assess parasite antigen burden during infection in the 3 months prior to the immunology blood draw, parasite density (log transformed) was calculated from blood-smear of most recent infection.

To assess relationships between cross-sectional CD4 T-cell responses and the prospective odds of infection measured at repeated, quarterly routine assessments, the odds of any infection (defined as either LAMP positive or blood smear positive with or without symptoms) and the odds of symptoms when patent infected (defined as malaria when patent blood smear infection detected) were calculated using multilevel mixed-effects logistic regression, accounting for repeat measures within individuals and clustered on household to account for multiple children in each individual household. In multivariate analysis, odds ratios for infection risk were adjusted for age (linear), dMER (categorical 0–8, >8–40, >40–80, >80 mosquitos/household/day) and PCR positive infection at blood draw.

To assess relationships between CD4 T-cell responses and parasite burden during asymptomatic infection, the geometric mean parasite burden across all detected infections (LAMP positive and blood smear positive) during routine visits was calculated. Associations with CD4 T-cell response were assessed with multivariant logistical regression controlling for age and infection status at time of sampling.

## Results

### Study Cohort and Clinical Outcomes

We performed a cross-sectional study of 265 children (aged 6 months to 10 years) enrolled in a longitudinal observational cohort in Nagongera, Uganda (Table 1) (14). This region has very high *Pf* malaria transmission, with an estimated annual entomological inoculation rate (aEIR) of 310 bites per person year (15). The malaria epidemiology of this cohort has previously been described (14). Briefly, parasite prevalence is high, increasing from 0.15 at 6 months to >0.3 by 4 years of age. Malaria incidence peaks at approximately four cases/year at age 3, and then declines (Table 1; Figure S3A in Supplementary Material). Consistent with these prior results, at the time of sampling 29% (*n* = 77) of children in our study had asymptomatic *Pf* infection detectable by blood smear (“patent” infection), and an additional 11% (*n* = 29) had subpatent *Pf* infection detectable by PCR. Thus, in total, 40% (*n* = 106) of our study population had asymptomatic infection (patent or subpatent) at time of sampling. Eighty percent (*n* = 211) of children had a blood smear positive parasite infection in the 3 months prior to sampling, at which the median log_10_ parasite density was 3.9 parasites/μL, IQR 3.3–4.6. Parasite density during recent infection correlated inversely with age (rho = –0.52, *p* < 0.0001, Figure S3B in Supplementary Material).

Blood was drawn for immunological analysis at a single cross-sectional time point, after which children were followed for 1 year, and the odds of parasite infection (patent and subpatent) and symptomatic malaria were assessed during quarterly clinic visits (Table 1). Increasing age was associated with increased odds of any infection (subpatent LAMP positive or patent blood smear positive with or without symptoms, OR 1.17, 95% 1.08–1.27, *p* < 0.001, Table S1 in Supplementary Material), consistent with prior reports in this cohort (14). For every increased year of age, there was a 15% reduced odds of malaria (OR 0.85, 95% CI 0.80–0.92 *p* < 0.001), consistent with the gradual development of clinical immunity. In order to control for the confounding factor of heterogeneity in exposure to *Plasmodium* transmission vectors in protection analyses, household mosquito counts were used to calculate an individual dMER in the year following blood draw (median 27.3 IQR 16.5–37.1 Anopheles/house/day, Figure S3C in Supplementary Material). After controlling for age and clustering by household, dMER was strongly associated with an increased odds of infection in the year following blood draw (OR 7.6, *p* = 0.007 for dMER > 80 compared with dMER 0- < 8, Table S1 in Supplementary Material), suggesting that this measure was able to account for some heterogeneity of exposure within the cohort.

### Asymptomatic Parasitemia Associated With a Lower Frequency of Circulating *Pf*-Specific CD4 T Cells

**Figure 1:**
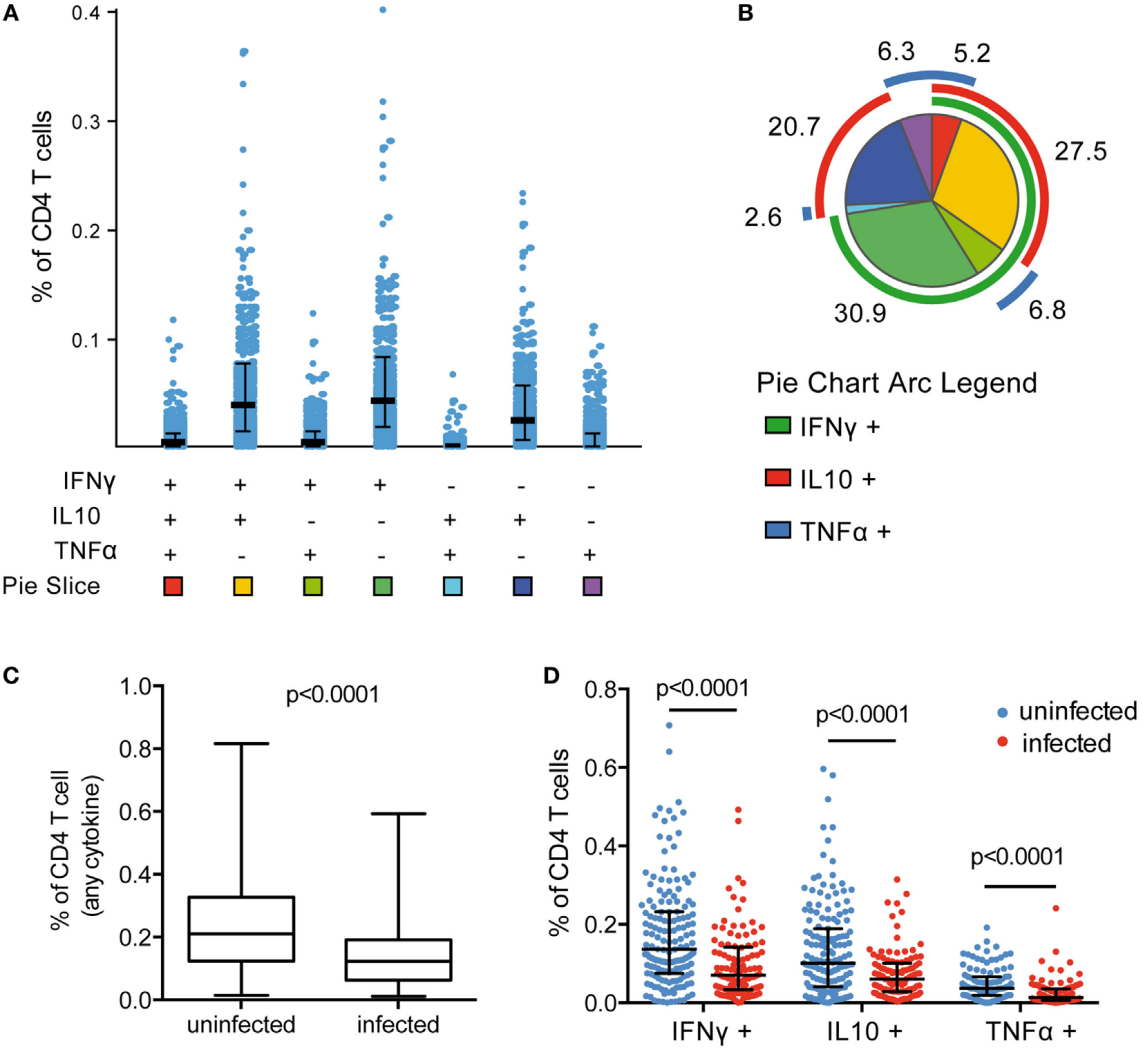
Frequency of *Pf* CD4 T-cell response reduced during asymptomatic infection. **(A)** *Pf*-specific CD4 T cells producing IFNγ, IL10, and TNFα were measured in 265 children *via* flow cytometry following incubation of PBMCs with parasite-infected RBCs. Responses were analyzed by Boolean gating in SPICE. **(B)** The proportion of CD4 T cells producing each cytokine combination was calculated as the percentage of the total parasite-specific CD4 T cells. A pie graph depicting the average proportion of these subsets among all children was generated in SPICE [pie slice colors correspond to the squares at the bottom of **(A)**]. Mean proportion of each response is indicated. **(C,D)** Current infection with *P. falciparum* was assessed *via* PCR. In currently infected children (*n* = 106), the total frequency of cytokine-producing CD4 T cells and IFNγ-, IL10-, and TNFα-producing CD4 T cells were all reduced compared with uninfected children (*n* = 159). Median and IQR are indicated by black bar and whiskers, with *p*-values for Wilcoxon rank sum test. IFNγ, interferon gamma; IL10, interleukin 10; PBMCs, plasma blood mononuclear cells; PCR, polymerase chain reaction; RBCs, red blood cells; TNFα, tumor necrosis factor alpha.

*Plasmodium falciparum*-specific CD4 T-cell production of IFNγ, IL10, and TNFα was measured by flow cytometry following stimulation of PBMCs with *Pf*-infected RBCs. Responding cells were analyzed as the frequency of total CD4 T cells. All individuals exhibited a measurable CD4 T-cell response to malaria, and among cytokine-producing CD4 T cells, on average 58% were monofunctional (produced a single cytokine), 36% coproduced two cytokines, and 4% coproduced all three cytokines. CD4 T cells producing IFNγ, IL10, or both, dominated the response (Figures 1A,B), consistent with previous observations (7–9). In children with asymptomatic infection (patent or subpatent) at time of blood draw, we observed a lower frequency of total *Pf-*specific CD4 T cells (Figure 1C), and of CD4 T cells producing any of the three cytokines (IFNγ, IL10, and TNFα), as well as all multi-cytokine-producing combinations defined by Boolean gating (Figure 1D; Figure S4A in Supplementary Material). The lower frequency of *Pf*-specific CD4 T cells among parasitemic children remained significant in multivariate analysis after controlling for both household levels of mosquito exposure (dMER) and age (Table S2 in Supplementary Material). There was no difference in the frequency of total CD4 T cells, as a percent of CD3 T cells (*p* = 0.51, Figure S5A in Supplementary Material) or as a percent of total lymphocytes (*p* = 0.71, Figure S5B in Supplementary Material). This finding is consistent with the hypothesized trafficking of *Pf-*specific CD4 T cells to inflamed sites during active parasite infection (19, 20). The composition of the *Pf-*specific response was also investigated by calculating the proportion of each cytokine-producing combination among the total *Pf-*specific CD4 T-cell population. All subsets of CD4 T-cell responses were reduced during *Pf* infection; hence, there was no difference in the proportion of these subsets between infected and uninfected children after controlling for age (Figure S4B in Supplementary Material).

### Coproduction of IL10 by *Pf*-Specific IFNγ-Producing CD4 T Cells Declining with Age

**Figure 2:**
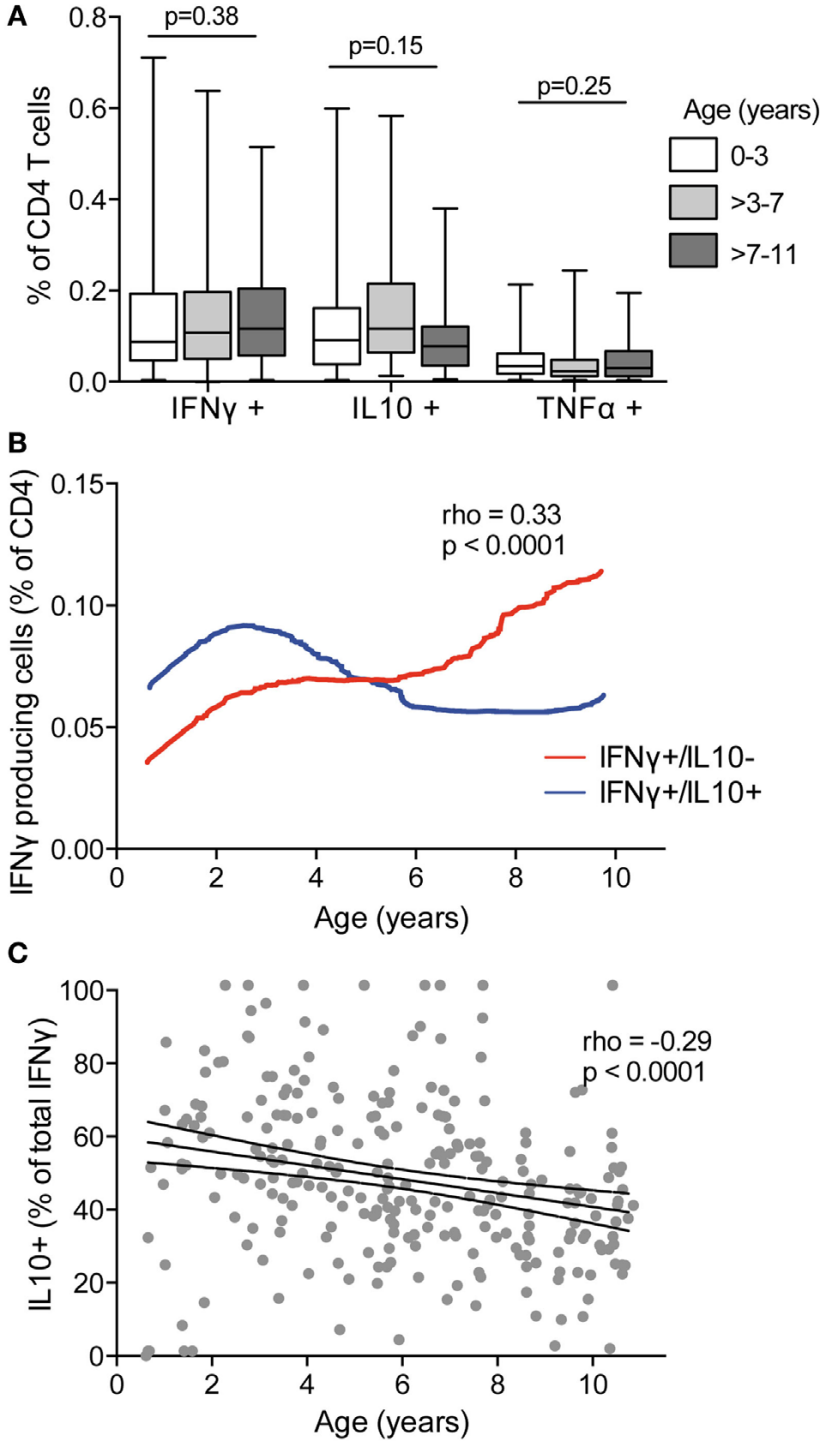
IFNγ in the absence of IL10 increasing with age and decreasing with IFNγ/IL10 coproduction. **(A)** The frequencies of total CD4 T cells producing IFNγ, IL10, or TNFα were compared between children aged 0 and 3 years (*n* = 46), >3–7 (*n* = 121), and >7–11 (*n* = 98). There was no difference in the frequencies of responding cells between age groups. Kruskal–Wallis comparisons across age groups for each cytokine are indicated. **(B)** The frequencies of CD4 T cells producing subsets of cytokines were analyzed by Boolean gating. IFNγ^+^/IL10^−^ with or without TNFα (IFNγ^+^/IL10^−^/TNFα^−^, and IFNγ^+^/IL10^−^/TNFα^+^) and IFNγ^+^/IL10^+^ with or without TNFα (IFNγ^+^/IL10^+^/TNFα^−^, and IFNγ^+^/IL10^+^/TNFα^+^) are collapsed for simplicity. Data are the lowess smoothed data with age, Spearman’s is for IFNγ^+^/IL10^−^ responses. **(C)** The proportion of the total IFNγ response that was coproducing IL10 was calculated as follows: $\frac{\text{\% CD4 Tcells producing IFNγ and IL10}(\text{with or without TNFα})}{\text{\% CD4 Tcell producing IFNγ}(\text{alone or in combination})}$. Rho and *p* for Spearman’s correlation with age is indicated. IFNγ, interferon gamma; IL10, interleukin 10; TNFα, tumor necrosis factor alpha.

Several murine and human studies have suggested a role for T cells producing the canonical Th1 cytokines IFNγ and TNFα in clearance of parasite infection, whereas T-cell production of IL10, often in combination with IFNγ, has been postulated to prevent immunopathology at the expense of parasite clearance [reviewed in Ref. (3)]. To examine how production of these cytokines by *Pf*-specific CD4 T cells changes with age, we compared children in three age strata (0–3, >3–7, and >7–11 years) spanning the age range during which clinical immunity to malaria typically emerges. We found no difference in the frequencies of total IFNγ-, IL10-, and TNFα-producing CD4 T cells across the different age strata (Figure 2A). However, when CD4 T-cell functional subsets were defined based on specific mono- or multifunctional subsets, several age-based trends became apparent. With increasing age, there was an increase in the frequency of *Pf-*specific CD4 T cells that produced IFNγ (with or without TNFα) but lacked IL10 production (rho = 0.33, *p* = 0.001), whereas the frequency of IFNγ^+^/IL10^+^ coproducing cells increased from 6 months to 3 years, and then declined (age < 3 rho = 0.41 *p* = 0.005 and age ≥ 3 rho = –0.17, *p* = 0.01, Figure 2B). In parallel, among IFNγ-producing CD4 T cells, the proportion that coproduced IL10 declined across all ages, from 6 months to 10 years (rho = –0.29, *p* < 0.001, Figure 2C). These associations remained significant after accounting for current parasite infection and dMER (adjusted regression Coeff. –1.9, *p* = 0.001). There was no association between age and IL10 or TNFα monofunctional CD4 T cells.

### IL10 Production by CD4 T Cells Associated With Recent High-Density Parasite Infection

**Figure 3:**
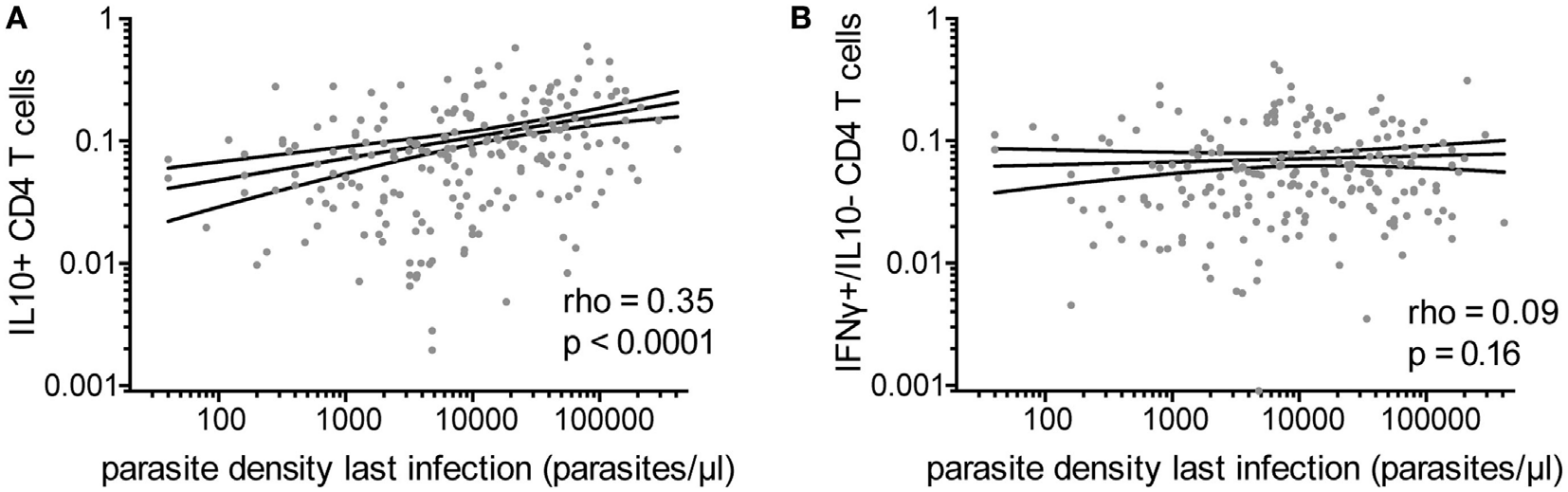
*Pf*-specific IL10^+^ CD4 T cells associated with parasite density during last infection. For children with a documented plasmodial infection in the preceding 3 months (*n* = 213), the association between the parasite density (parasites/μL) during the most recent infection and **(A)** the frequency of total *Pf*-specific IL10^+^ CD4 T cells or **(B)** IFNγ^+^/IL10^−^ cells are shown. IFNγ, interferon gamma; IL10, interleukin 10; *Pf, Plasmodium falciparum*.

Because IFNγ/IL10 CD4 T-cell responses have been associated with high antigen burden in other systems (21, 22), we assessed the relationship of *Pf*–specific responses and parasite density during the most recent infection (within 3 months prior to blood draw). Among the 80% of children who had a parasite infection in the preceding 3 months (with or without symptoms), there was a highly significant positive correlation between the frequencies of *Pf-*specific total IL10 producing CD4 T cells and parasite density during infection (rho = 0.35, *p* < 0.0001, Figure 3A). This relationship was seen with both IFNγ/IL10 coproducing and IL10 monofunctional (IFNγ^−^/IL10^+^) (Spearman’s rho = 0.37 *p* < 0.0001 and rho = 0.2 *p* = 0.004, respectively). Parasite density was not associated with the frequency of CD4 T cells producing IFNγ without IL10 (Figure 3B) or TNFα responses (monofunctional or in combination with IFNγ). The total IL10 response and frequency of IFNγ/IL10 coproducing cells, but not IL10 single-producing cells, were weakly associated with malaria incidence in the preceding year (rho = 0.14, *p* = 0.02, rho = 0.16, *p* = 0.01 and rho = 0.026, *p* = 0.67, respectively), consistent with prior reports (8). We did not observe any associations between household mosquito exposure (dMER) and IL10 production by *Pf*-specific CD4 T cells. While younger children tended to have higher parasite densities (Figure S1B in Supplementary Material), age did not fully explain the relationship between IL10-producing cells and parasite density during recent infection, as the association with parasite density and total IL10 or IFNγ/IL10 coproducing cells remained significant after controlling for age, dMER, and current infection (adjusted regression coefficient for total IL10 0.15, *p* < 0.001 and for IFNγ^+^/IL10^+^ cells 0.16, *p* < 0.001).

### *Pf*-Specific IL10-Producing CD4 T Cells Expressing BLIMP-1

**Figure 4:**
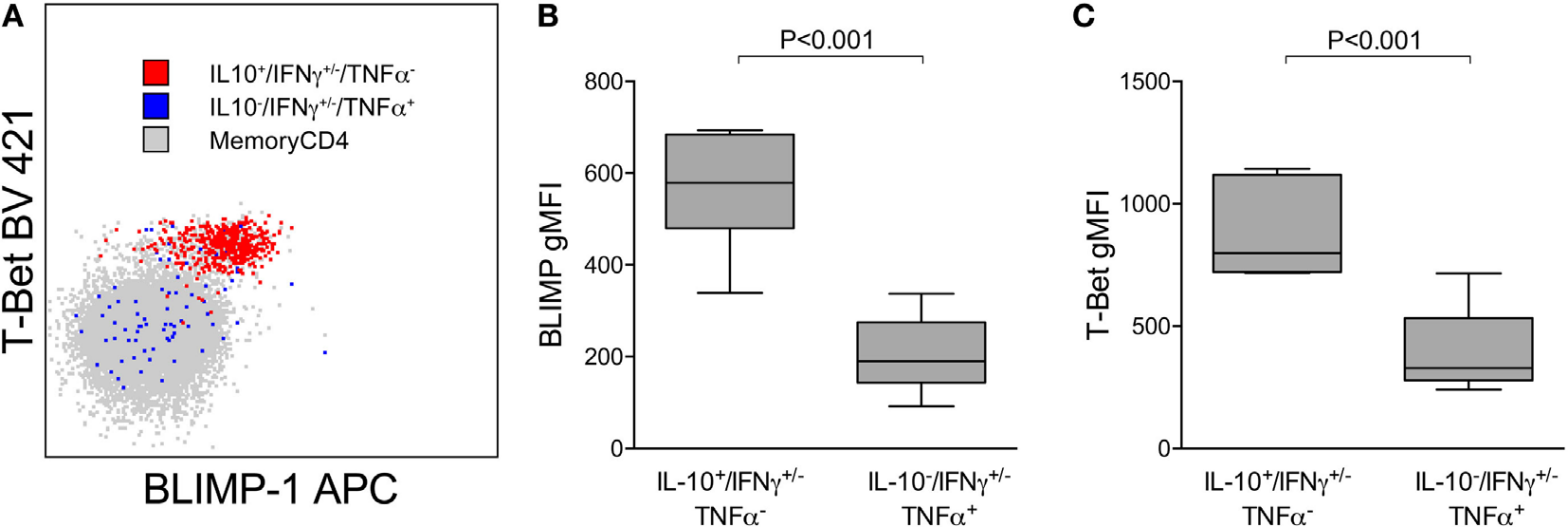
IL10^+^ CD4 T cells expressing Tbet(hi) and BLIMP-1. **(A)** Representative plot showing expression of the transcription factors T-bet and BLIMP-1 on *Pf-*specific responses production IL10 (IL10^+^/IFN^+/−^/TNFα^−^) or TNFα (IL10^−^/IFN^+/−^/TNFα^+^) within one individual. Relative T-bet **(B)** and BLIMP-1 **(C)** expression on *Pf-*specific responses production IL10 (IL10^+^/IFN^+/−^/TNFα^−^) and TNFα (IL10^−^/IFN^+/−^/TNFα^+^). Shown are data from 15 independent individuals. gMFI, geometric mean fluorescence intensity; IFNγ, interferon; IL10, interleukin 10; TNFα, tumor necrosis factor alpha.

We have previously shown that *Pf*-specific IFNγ/IL10 coproducing cells express the canonical Th1 transcription factor T-bet and are not FoxP3^+^ regulatory T cells (8). IL-10 in Th1 CD4 cells may be expressed in part to limit inflammation, at the cost of impaired pathogen clearance (23). Recent reports have also shown that the transcriptional regulator BLIMP-1 is required to program Th effectors into IL-10 producers in murine models of chronic infection (24, 25) [including *Plasmodium* (13)]. We assessed T-bet and BLIMP-1 expression among *Pf*-cytokine-producing cells (Figure 4A) and found that levels of T-bet were significantly higher on IL10 producing cells than on IFNγ or TNFα cells that did not produce IL10 (Figure 4B). Furthermore, BLIMP-1 was highly expressed among *Pf*-specific IL10-producing CD4 T cells, but not on IFNγ or TNFα cells that did not produce IL10 (Figure 4C). Taken together, these data suggest that IL10 production by *Pf*-specific CD4 T cells is associated with high-parasite antigen burden in children heavily exposed to malaria, is independently influenced by age, and may be induced through expression of the transcriptional regulator BLIMP-1, as recently reported in murine models (13).

### Relationship of the CD4 T-Cell Response to Protection from *Pf* Infection and Clinical Malaria

#### Prospective Protection from *Pf* Infection

**Figure 5:**
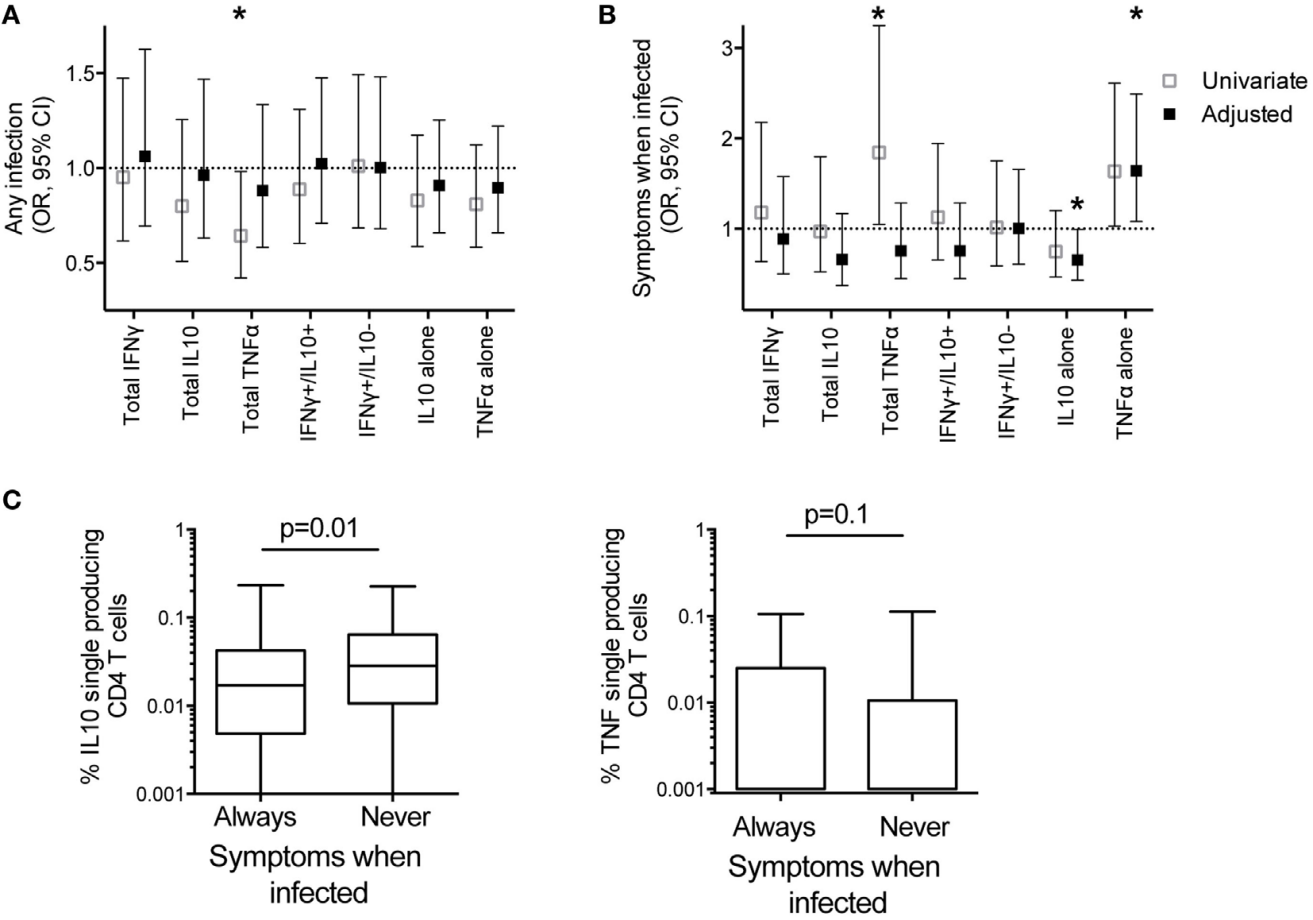
IL10 single-producing CD4 T cells associated with protection from symptomatic malaria. The associations between frequencies of *Pf-*specific total CD4 T cells producing IFNγ, IL10, TNFα, or specific subsets of responses (IFNγ^+^/IL10^+^, and IFNγ^−^/IL10^+^ with or without TNFα, and IL10^+^ and TNFα^+^ single-producing CD4 T cells) with clinical outcomes in the year following blood draw were calculated by generalized estimate equations with robust SEs accounting for repeated measures within patients; **(A)** Odds ratios for associations with any infection (LAMP positive or blood smear positive). Data are univariate or adjusted for age, mosquito exposure (dMER), and bloodsmear and PCR positive infection at time of sampling, clustered on household. **(B)** Odds ratios for associations with probability of symptomatic malaria given patent infection (blood smear positive). Data are univariate, or adjusted for age and bloodsmear and PCR positive infection at blood draw. *p < 0.05. **(C)** The frequencies of *Pf*-specific IL10^+^ single-producing CD4 T and TNFα^+^ single-producing CD4 T cells between children who had at least one blood smear positive infection, who were always symptomatic when infected (*n* = 102), compared with those who were never symptomatic (*n* = 26) when infected in the year following blood draw *p*-values based on Wilcoxon rank sum test). IFNγ, interferon gamma; IL10, interleukin 10; *Pf, Plasmodium falciparum*; dMER, daily mosquito exposure rate; LAMP, loop-mediated isothermal amplification; PCR, polymerase chain reaction; TNFα, tumor necrosis factor alpha.

Mounting evidence suggests that the immune mechanisms underlying protection from *Pf* infection (or restriction of parasite growth) may be distinct from those that mediate “clinical immunity,” or protection from symptomatic malaria. To investigate the role of cytokine-producing CD4 T cells in both aspects of naturally acquired immunity, we first analyzed the relationship between *Pf-*specific CD4 T-cell responses and protection from any *Pf* infection during 1 year of prospective follow-up. Subjects were assessed during routine quarterly visits for infection detected by blood smear (with or without symptoms) or by the sensitive LAMP assay (16). In univariate analysis, a higher total frequency or proportion of total TNFα-producing CD4 T cells was associated with a lower risk of infection (any infection, blood smear, or LAMP detected; *p* = 0.04 and 0.007, Figure 5A; Table S3 in Supplementary Material). However, after adjusting for age, dMER, and infection status at sampling (PCR and/or blood smear positive infection), these relationships were not significant (*p* = 0.55 and 0.26; Table S3 in Supplementary Material). In contrast, the proportion of cells producing IFNγ without IL10 was associated with a trend to *increased* prospective risk of infection in univariate analysis, but this was not significant following adjustment for dMER and other covariates (OR 1.01 for a 1% increase in IFNγ production in the absence of IL10, *p* = 0.06, Table S3 in Supplementary Material). There was no association between the frequency of total IFNγ, total IL10, nor other subsets of CD4 T-cell responses and prospective risk of infection (Figure 5A; Table S3 in Supplementary Material). In summary, among highly exposed children, all of whom had a measurable *Pf*-specific CD4 T-cell response, neither the frequency of these cells nor their cytokine production profiles correlated with prospective protection from infection after adjustment for household exposure intensity.

#### Prospective Protection from Clinical Malaria Once Infected

Although not all infections result in symptomatic malaria and asymptomatic infections increase with age and prior exposure, we separately analyzed the conditional probability that an individual developed symptomatic malaria, given that s/he had a patent blood smear positive infection. After controlling for age and infection at time of sampling (PCR and/or blood smear positive infection), higher frequencies of *Pf-*specific monofunctional IL10 CD4 T cells were associated with a *decreased* odds of symptoms, given patent infection (adjusted OR 0.66 95% CI 0.43–0.99, *p* = 0.05, Figure 5B; Table S4 in Supplementary Material. In contrast, the frequency of *Pf-*specific monofunctional TNFα CD4 T cells was associated with an *increased* odds of developing symptomatic malaria, given patent *Pf* infection (OR 1.64 95% CI 1.08–2.49, *p* = 0.02, Figure 5B; Table S4 in Supplementary Material). Consistent with these findings, in the subsequent year, children whose patent infections were all asymptomatic had increased frequencies of *Pf*-specific monofunctional IL10 CD4 T cells (*p* = 0.01) compared with children who always experienced malaria when infected (total children *n* = 128, analysis includes non-routine visits to study clinic) (Figure 5C). While there was also a trend to decreased frequencies of *Pf-*specific monofunctional TNFα CD4 T cells in children who always were asymptomatic when infected, this association was not statistically significant (*p* = 0.1). There was no association between the frequency of other functional subsets and the odds of symptoms given patent infection (Figure 5B).

## Discussion

Prior studies in both humans and animal models support an important role for CD4 T cells in mediating immunity to malaria [reviewed in Ref. (3)]. However, the factors influencing functional differentiation of *Pf-*specific CD4 T cells and the role of specific cytokine-producing subsets in protective immunity remain poorly understood. Here, in a large cohort of children aged 6 months to 10 years, we found that recurrent and/or high-density *Pf* parasitemia was associated with an IL10-dominant CD4 T-cell phenotype, particularly in younger children. *Pf-*specific IL10 production from CD4 T cells, specifically in the absence of IFNγ coproduction, was associated with a reduced likelihood of febrile malaria upon *Pf* infection. In contrast, our data suggest that monofunctional TNFα CD4 T cells may be associated with a higher likelihood of symptoms once infected.

Our data bolster the existing evidence that protection from *Pf* infection and protection from clinical malaria are mediated by distinct immunological processes. In our cohort, the prospective risk of infection increased between 0 and 10 years of age as reported previously (26), possibly due to increasing body surface area (27), behavioral factors such as bed net usage (28) and/or treatment patterns due to increasing immunity. In contrast, the risk of symptomatic malaria and parasite density during infection declined over this age range. These observations are in agreement with those of Tran et al., who reported no difference in time-to-infection by age among seasonally exposed Malian adults and children (2). Taken together, these findings support the notion that the age-associated clinical immunity observed among residents of endemic regions does not confer sterile protection against *Pf* parasitemia. However, parasite density declined notably over this age interval, indicating the emergence of an adaptive immune response capable of restricting parasite growth and limiting antigen burden. Identifying immune correlates of protection from *Pf* infection and clinical malaria in naturally exposed populations has been difficult for several reasons, including the challenge of accounting for heterogeneity in exposure to infected mosquitoes. Our cohort has the distinct advantage of having a rich longitudinal clinical dataset as well as measures of mosquito exposure both prior to and following the blood draw, enabling evaluation of both the determinants of the CD4 response and correlates of prospective protection from both parasitemia and clinical malaria.

Consistent with our prior observations, the majority of *Pf*-specific CD4 T cells in heavily exposed Ugandan children produced the immunoregulatory cytokine IL10 (7–9). We show that CD4 IL10 production is associated with high-parasite antigen burden, consistent with other infections as well as non-infectious disease models (21, 29). Secretion of IL10 by CD4 T cells may attenuate pathogen-induced immunopathology by both directly inhibiting T-cell cytokine production and proliferation, and limiting the function of antigen presenting cells. We and others have shown that this IL10 is produced largely by CD4 T cells expressing the Th transcription factor Tbet, many of which coproduce IFNγ. Our prior data suggest that these cells appear consistent with “early effector memory” cell phenotype (CD45RA^−^, CCR7^−^, CD27^+^) (8), whereas *Pf-*specific CD4 T cells producing inflammatory cytokines IL2 and TNFα are more consistent with “central memory” phenotypes (CD45RA^−^CCR7^+^) (7). Although IL-10 can be produced by a variety of CD4 T-cell subsets, IL-10 production by using the Th1 subset has been shown to be required for attenuation of morbidity and immune-mediated pathology during primary murine malaria infection (30, 31) and other protozoal infections (11, 12). Here we show that *Pf-*specific IL10-producing CD4 T cells in children express the transcription factor BLIMP-1, a transcriptional regulator recently shown to be critically dependent for IL-10 production from murine “T_R_1 cells” (13), further supporting the notion that IL10-producing *Pf-*specific CD4 T cells are Th1 origin. In contrast *Pf-*specific CD4 T cells producing TNFα do not express BLIMP-1 and express significantly lower levels of Tbet.

In our cohort, it was specifically monofunctional IL10 CD4 T cells that were associated with a reduced risk of symptomatic malaria following *Pf* infection; high frequencies of these cells were significantly associated with reduced odds of symptomatic malaria when infection. This is consistent with studies from children who are seasonally exposed to malaria, where IL-10 production following *in vitro* stimulation of PBMCs with malaria antigens was higher among children who persistently harbored parasites through the dry season, but not in children lacking recent parasite exposure (10). Furthermore, a recent study indicated that patients with severe malaria had reduced proportions of IL10 single-producing CD4 T cells compared with those with uncomplicated malaria, while the proportions of IFNγ single-producing or IFNγ/IL10 coproducing CD4 T cells were similar between disease phenotypes (32). *In vitro* stimulation studies have shown that Th1 IFNγ CD4 T cells can switch to IFNγ/IL10 coproducing, and then to IL10 single-producing CD4 T cells with repeated stimulation (33). In our cohort, both IFNγ/IL10 coproducing and IL10 single-producing subsets were associated parasite density during recent infection, while only IL10 single-producing responses were associated with protection from symptomatic disease. Together, these data suggest that a final switch from IFNγ to IL10 production among malaria-specific CD4^+^ T cells may play a role for in mediating tolerance to malaria. Importantly, exploitation of this pathway has recently been shown to be effective as antigen-specific immunotherapy for autoimmune disease (34).

Our data also indicate that biological age influences CD4 T-cell production of IL10 *independently* of antigen burden, with younger children biased toward a more IL10-skewed CD4 T-cell response, suggesting an inherent age-related developmental difference in the cellular immune response (35). In areas of high malaria transmission, untangling the influence of age and exposure on the immune response is complicated by the colinearity of age and cumulative parasite exposure. Here we used detailed clinical and entomological data to show that age and exposure independently influence the *Pf-*specific CD4 T-cell response. Innate immune responses to T-like receptor stimulation are dominated by regulatory cytokines including IL10 in early life, and the production of cytokines that support Th1 CD4 T-cell development are one of the last to reach adult levels (36–38). This regulatory bias in early life may in part help to explain observations made among Indonesian migrants moving from areas with little or no malaria to high-transmission regions where young children with their first malaria infection were far *less* susceptible to severe malaria and death than adults, but adults developed immunity to mild malaria more rapidly than children (39–42). Despite the age-associated decline in IL10 production, even the oldest children in our cohort exhibited higher frequencies of IL10 CD4 T cells and lower frequencies of TNFα production by CD4 T cells than adults at the same study site (9), which may be related to continued immune maturation and/or to the much lower rates of patent *Pf* parasitemia among adults in this cohort (26).

Our data suggest that in contrast to IL10, *Pf-*specific monofunctional CD4 cells producing TNFα may be associated with a *higher* risk of clinical disease once infected. While the strength and significance of this relationship was less clear in the current study, it is supported by our previous finding that prevalence of asymptomatic parasite infection was reduced in 4-year-old children with high frequencies of *Pf*-specific TNFα-monofunctional CD4 T cells (8). In the present cohort, TNFα-producing CD4 cells were also associated with protection from infection in univariate analysis, but this association was not significant after controlling for exposure intensity and age. This result suggests that TNFα production from CD4 T cells may be associated with low parasite exposure, highlighting the importance of accounting for heterogeneous exposure to *Pf-*infected mosquitoes in cohort studies examining correlates of prospective infection risk. Together our findings underscore the delicate balance of CD4 T-cell responses required to mediate protection from infection and disease. We did not find any associations between CD4 production of IFNγ and protection from malaria, consistent with our previous studies in this high-transmission setting (7, 8). This is in contrast to previous reports finding such associations when looking at IFNγ responses *via* methods that assess total cytokine from PBMCs (4). These differing results reflect important differences between the use of multi-parametric flow cytometer to assess individual subsets of CD4 T-cell responses verses methods that evaluate global assessments of cytokine production which are unable to distinguish between IFNγ from CD4 T cells, with that from other sources—for example from γδ T cells where IFNγ production may have a differing role in protection and disease (43–45).

This study has several limitations inherent in its cross-sectional sampling of individuals, including the inability to assess the functional differentiation of CD4 T-cell responses within individuals across time. In addition, we measured CD4 production of only three cytokines, but other CD4 cytokine-producing phenotypes may also be important (7, 46). Furthermore, although we observed significant heterogeneity in environmental exposure to *Pf* based on entomological measures, the vast majority of cohort participants had very high levels of exposure intensity, such that even children with the lowest levels of exposure in this study cohort had very high exposure relative to most malaria-endemic communities. In a study comparing children and adults from the current study site to those in a nearby low-transmission site, we have previously shown that exposure strongly influences the functional phenotype of *Pf-*specific CD4 T-cell responses (9). We did not observe marked differences based on exposure intensity in the current study, perhaps due to “saturated” mosquito exposure. Hence, our findings may not be applicable to other settings with lower transmission intensity. Lastly, while adjustment for entomological exposure helped to limit confounding by exposure intensity, it is possible that associations between *Pf*–specific CD4 T-cell profiles and prospective clinical outcomes may be driven by the impact of antigen burden on CD4 functional phenotype, rather than CD4-mediated immune protection.

In conclusion, in a large cohort of naturally exposed children, we have identified factors influencing the cytokine production profiles of *Pf-*specific CD4 T cells, with both exposure and age being independently associated with IL10 CD4 T-cell responses. Monofunctional IL10 CD4 T cells were associated with high antigen burden and/or repeated infections, and were associated with protection from symptoms once infected. These findings elucidate important factors that influence the development of *Pf* -specific CD4 T cells and their roles in human immunity to malaria.

## Ethics Statement

This study was carried out in accordance with the recommendations of Uganda National Council of Science and Technology and the institutional review boards of the University of California, San Francisco, and Makerere University, with written informed consent from all adult subjects or parent/guardian of all study participants. All subjects gave written informed consent in accordance with the Declaration of Helsinki. The protocol was approved by the institutional review boards of the University of California, San Francisco (institutional review board number 11-05995; reference number 067647), and Makerere University.

## Author Contributions

Conceptualization: MB, PJ, and MF. Methodology: MB, PJ, MF, and KB. Software: BG. Formal analysis: MB, PJ, BG, GD, and MF. Investigation: MB, KB, TM, HV, LF, and AS. Contributed reagents/materials/analysis tools: FN, KN, SW, ES, JR, BG, EA, and MK. Wrote the paper: MB, PJ, LF, BG, GD, and MF. All authors contributed to editing and revising manuscript.

## Conflict of Interest Statement

The authors declare that the research was conducted in the absence of any commercial or financial relationships that could be construed as a potential conflict of interest.
