# Supplementary material for: The Development of Plasmodium falciparum-Specific IL10 CD4 T Cells and Protection from Malaria in Children in an Area of High Malaria Transmission
Source: Front Immunol. 2017 Oct 19;8:1329. doi: 10.3389/fimmu.2017.01329 (PMC5653696; doi:10.3389/fimmu.2017.01329)
Supplement: Supplementary file 1 [file Table_1.DOCX]

Supplementary Material

The Development of *Plasmodium falciparum*-specific IL10 CD4 T Cells and Protection From Malaria in Children in an Area of High Malaria Transmission

**Michelle J. Boyle, Prasanna Jagannathan, Katherine Bowen, Tara I. McIntyre, Hilary M. Vance, Lila A. Farrington, Alanna Schwartz, Felistas Nankya, Kate Naluwu, Samuel Wamala, Esther Sikyomu, John Rek, Bryan Greenhouse, Emmanuel Arinaitwe, Grant Dorsey, Moses R. Kamya, Margaret E. Feeney^*^**

*** Correspondence:** Margaret Feeney: Margaret.feeney@ucsf.edu

# Supplementary Tables

**Supplementary Table 1: Associations of age and daily mosquito exposure rate with odds of any infection in the year following blood draw**

|  | Probability of infection | | | |  | | | |
| --- | --- | --- | --- | --- | --- | --- | --- | --- |
|  | Univariate | | | | Multivariate^1^ | | | |
|  | OR | 95% CI | | *P* | OR | 95% CI | | *P* |
| Age | 1.17 | 1.08 | 1.27 | **<0.001** | 1.18 | 1.1 | 1.28 | **<0.001** |
| dMER ^2^ ref 0-8 |  |  |  |  |  |  |  |  |
| >8-40 | 2.17 | 0.9 | 5.28 | 0.09 | 2.54 | 1.1 | 5.88 | 0.03 |
| >40-80 | 2.71 | 0.96 | 7.67 | 0.06 | 3.36 | 1.25 | 9.04 | 0.02 |
| >80 | 6.14 | 1.28 | 29.4 | 0.02 | 7.63 | 1.73 | 33.61 | 0.01 |

1. For probability of infection, associations with odds of any infection at routine visit in the year following blood draw (LAMP positive and blood smear positive with or without symptoms) was calculated with multi-level mixed effects logistic regression models. Multivariate analysis includes age, dMER (clustered on household) and infection at time of blood draw, which is a confounder of protection analysis.
2. dMER, daily mosquito exposure rate, female Anopheles mosquitos/house/day.

**Supplementary Table 2: Magnitudes of CD4 T cell cytokine producing cells are reduced during asymptomatic infection, independently of age**

|  | Univariate | | | Controlled by age, dMER | | |
| --- | --- | --- | --- | --- | --- | --- |
| (log10) | Coef | 95% CI | P | Coef | 95% CI | P |

| Total cytokine | -0.23 | -0.33 | -0.13 | **<0.001** | -0.26 | -0.36 | -0.17 | **<0.001** |
| --- | --- | --- | --- | --- | --- | --- | --- | --- |
| Total IFNγ | -0.25 | -0.37 | -0.13 | **<0.001** | -0.31 | -0.41 | -0.20 | **<0.001** |
| Total IL10 | -0.21 | -0.33 | -0.09 | **0.001** | -0.23 | -0.33 | -0.12 | **<0.001** |
| Total TNFα | -0.34 | -0.48 | -0.21 | **<0.001** | -0.35 | -0.51 | -0.2 | **<0.001** |

The impact of current asymptomatic parasite infection on the magnitude of *P. falciparum* specific cytokine producing CD4 T cells was analysed by regression analysis. Multivariate analysis included age, dMER (clustered on household).

**Supplementary Table 3: Odds ratios for the associations between CD4 T cell responses and any infection (LAMP or blood smear positive) during routine visits in year following blood draw.**

|  | Univariate | | | | Multivariate ^#^ | | | |
| --- | --- | --- | --- | --- | --- | --- | --- | --- |
| % CD4 T cells (log_10_) | OR | 95% CI | | *P* | OR | 95% CI | | *P* |
| Total IFNγ | 0.95 | 0.62 | 1.47 | 0.83 | 1.06 | 0.69 | 1.63 | 0.78 |
| Total IL10 | 0.78 | 0.51 | 1.26 | 0.33 | 0.93 | 0.63 | 1.47 | 0.86 |
| Total TNF | 0.64 | 0.42 | 0.98 | **0.04** | 0.88 | 0.58 | 1.33 | 0.55 |
| IFNγ^+^/IL10^+^ * | 0.89 | 0.60 | 1.32 | 0.55 | 1.02 | 0.71 | 1.48 | 0.90 |
| IFNγ^+^/IL10^-^ * | 1.01 | 0.68 | 1.49 | 0.96 | 1.00 | 0.68 | 1.48 | 0.99 |
| IL10 alone | 0.83 | 0.59 | 1.17 | 0.29 | 0.91 | 0.66 | 1.25 | 0.56 |
| TNFα alone | 0.81 | 0.58 | 1.12 | 0.21 | 0.90 | 0.66 | 1.22 | 0.48 |
| Proportion of responding CD4 T cells |  |  |  |  |  |  |  |  |
| Total IFNγ | 1.01 | 1.00 | 1.02 | 0.21 | 1.00 | 0.99 | 1.01 | 0.42 |
| Total IL10 | 1.00 | 0.99 | 1.01 | 0.35 | 1.00 | 0.99 | 1.01 | 0.59 |
| Total TNF | 0.98 | 0.97 | 1.00 | **0.007** | 0.99 | 0.98 | 1.01 | 0.26 |
| IFNγ^+^/IL10^+^ * | 1.00 | 0.98 | 1.01 | 0.59 | 1.00 | 0.99 | 1.01 | 0.97 |
| IFNγ^+^/IL10^-^ * | 1.01 | 1.00 | 1.02 | 0.06 | 1.01 | 0.99 | 1.02 | 0.39 |
| IL10 alone | 1.00 | 0.99 | 1.01 | 0.86 | 1.00 | 0.99 | 1.01 | 0.60 |
| TNFα alone | 0.98 | 0.97 | 1.00 | **0.007** | 0.99 | 0.98 | 1.01 | 0.26 |

* IFNγ^+^/IL10^+^ and IFNγ^+^/IL10^-^ responses include both TNFα^+^ and TNFα^-^ cells

# multivariate analysis is adjusted for age and infection (patent, blood smear detected and sub-patent, PCR detected) at time of sampling, and dMER in the year following blood draw by multi-level mixed effects models clustered on household.

**Supplementary Table 4: Odds ratios for the associations between CD4 T cell responses and symptomatic malaria occurring when infected with patent blood smear positive infection in year following blood draw.**

|  | Univariate | | | | Multivariate ^#^ | | | |
| --- | --- | --- | --- | --- | --- | --- | --- | --- |
| % CD4 T cells (log_10_) | OR | 95% CI | | *P* | OR | 95% CI | | *P* |
| Total IFNγ | 1.18 | 0.64 | 2.18 | 0.60 | 0.89 | 0.50 | 1.58 | 0.69 |
| Total IL10 | 0.97 | 0.53 | 1.80 | 0.93 | 0.66 | 0.37 | 1.17 | 0.16 |
| Total TNF | 1.85 | 1.05 | 3.25 | **0.03** | 0.76 | 0.45 | 1.28 | 0.30 |
| IFNγ^+^/IL10^+^ * | 1.13 | 0.65 | 1.94 | 0.67 | 0.76 | 0.45 | 1.28 | 0.30 |
| IFNγ^+^/IL10^-^ * | 1.02 | 0.59 | 1.75 | 0.96 | 1.01 | 0.61 | 1.66 | 0.99 |
| IL10 alone | 0.75 | 0.47 | 1.20 | 0.23 | 0.66 | 0.43 | 0.99 | **0.05** |
| TNFα alone | 1.64 | 1.03 | 2.61 | **0.04** | 1.64 | 1.08 | 2.49 | **0.02** |

* IFNγ^+^/IL10^+^ and IFNγ^+^/IL10^-^ responses include both TNFα^+^ and TNFα^-^ cells

# multivariate analysis is adjusted for age and infection (patent, blood smear detected and sub-patent, PCR detected) at time of sampling, clustered on household.
