# Supplementary material for: The Development of Plasmodium falciparum-Specific IL10 CD4 T Cells and Protection from Malaria in Children in an Area of High Malaria Transmission
Source: Front Immunol. 2017 Oct 19;8:1329. doi: 10.3389/fimmu.2017.01329 (PMC5653696; doi:10.3389/fimmu.2017.01329)
Supplement: Supplementary file 2 [file Presentation_1.PDF]

Supplementary Material

The Development of *Plasmodium falciparum*-specific IL10 CD4 T Cells and Protection From Malaria in Children in an Area of High Malaria Transmission

Michelle J. Boyle, Prasanna Jagannathan, Katherine Bowen, Tara I. McIntyre, Hilary M. Vance, Lila A. Farrington, Alanna Schwartz, Felistas Nankya, Kate Naluwu, Samuel Wamala, Esther Sikyomu, John Rek, Bryan Greenhouse, Emmanuel Arinaitwe, Grant Dorsey, Moses R. Kamya, Margaret E. Feeney*

*** Correspondence:** Margaret Feeney: Margaret.feeney@ucsf.edu

1 Supplementary Figures

1.1 Supplementary Figure S1

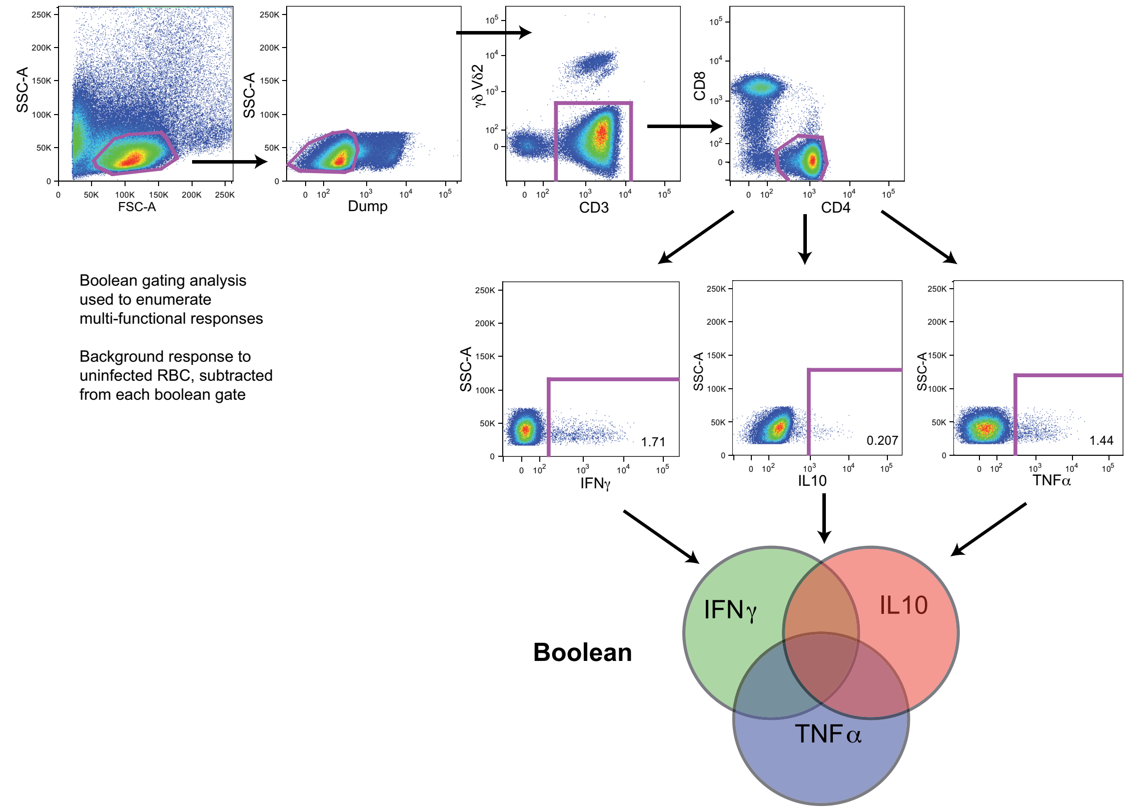

Supplementary Figure S1: Gating strategy for *Pf*-specific IFNγ, IL10 and TNFα CD4 T cell responses.

Gating strategy to identify CD4+ cytokine cells producing interferon γ (IFN-γ), interleukin 10 (IL-10), and tumor necrosis factor α (TNF-α) following stimulation with *Pf*–infected red blood cells (RBCs). Background responses (to uninfected RBCs) were subtracted from each Boolean gate.

1.2 Supplementary Figure S2

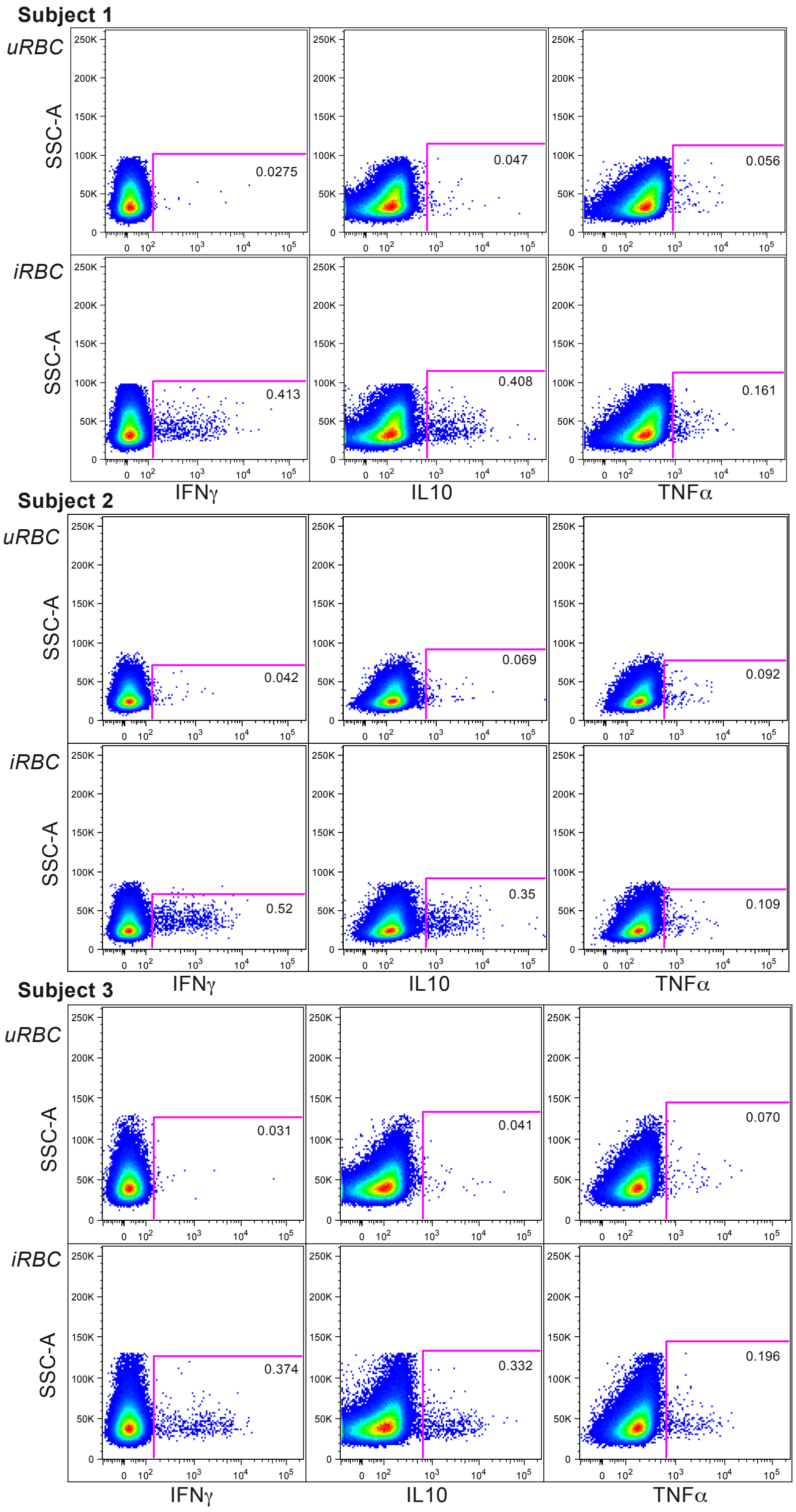

Supplementary Figure S2: Example cytokine production from uninfected and infected RBC stimulations. CD4+ cells producing interferon γ (IFN-γ), interleukin 10 (IL-10), and tumor necrosis factor α (TNF-α) following stimulation with uninfected (uRBC) and *Pf*–infected RBCs (iRBC).

1.3 Supplementary Figure S3

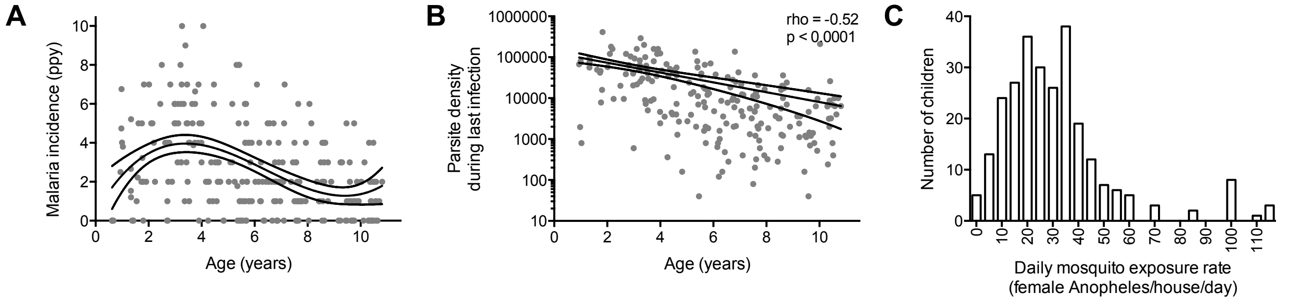

Supplementary Figure S3: Study cohort characteristics.
(A) The association between malaria incidence in the year prior to blood draw and age. **(B)** The association between parasite density at last infection (in children with a recorded patent blood smear positive infection in the last 3 months). Spearman rho and p indicated. **(C)** Distribution of daily mosquito exposure rate disease incidence in the year following blood-draw.

1.4 Supplementary Figure S4

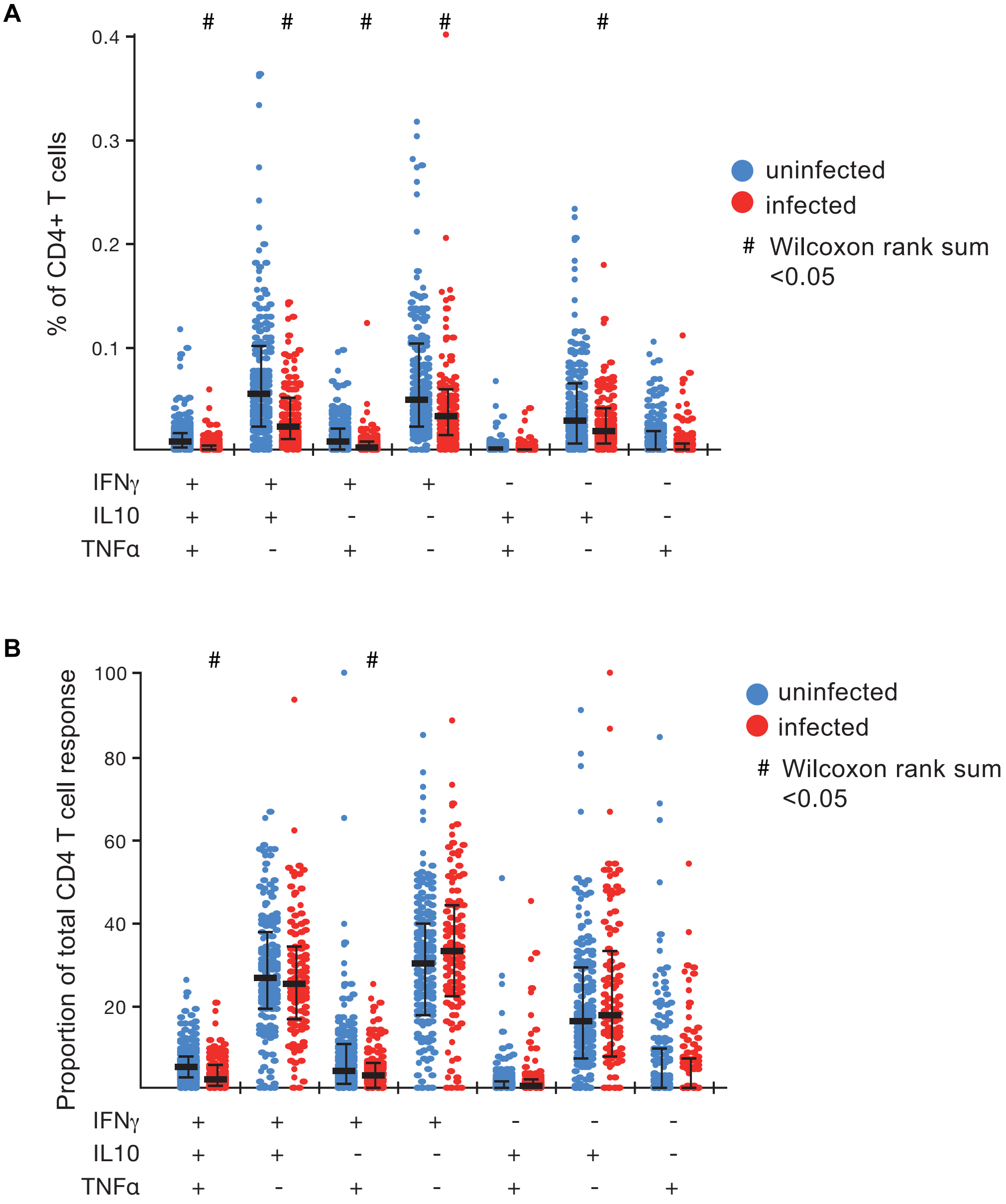

Supplementary Figure S4: Frequencies but not proportions of *P. falciparum* CD4 T cell response are reduced during asymptomatic infection.
P. falciparum CD4 T cell responses producing IFNγ, IL10 and TNFα were in 265 children measured via flow-cytometry following incubation of PBMCs with parasite infected RBCs. Responses were analyzed by Boolean gating in SPICE and responses compared between PCR positive and PCR negative children. **(A)** The frequencies of responses were reduced in currently infected children. **(B)** The proportion of each response as a percentage of total responding CD4 T cells was compared between infected and uninfected children. There was no difference between uninfected and infected after controlling for age. Median and IQR are indicated by black bar and whiskers.

1.5 Supplementary Figure S5

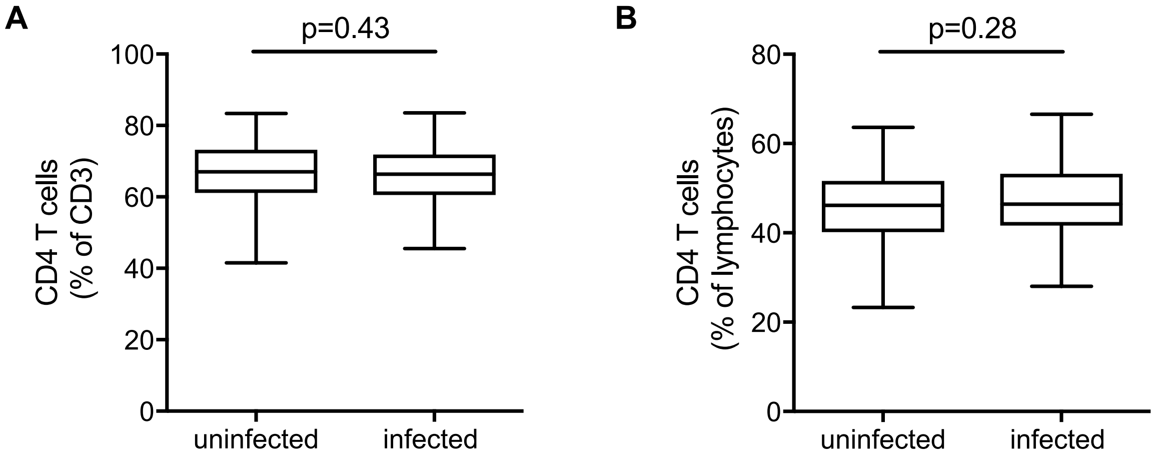

Supplementary Figure S5: Frequencies to total CD4 T cells show no difference between uninfected and infected children.
Total CD4 T cells gated as percent of CD3 T cells **(A)** or as a percent of total lymphocytes **(B)** were assessed in 265 children via flow-cytometry in PBMCs stimulated with iRBCs. There was no difference between uninfected and infected children. Median and minimum/maximum are indicated by black bar and whiskers. P is Mann-Whitney t-test.
